# Identification of *N^6^,N^6^*-Dimethyladenosine in Transfer RNA from *Mycobacterium bovis* Bacille Calmette-Guérin

**DOI:** 10.3390/molecules16065168

**Published:** 2011-06-21

**Authors:** Clement T.Y. Chan, Yok Hian Chionh, Chia-Hua Ho, Kok Seong Lim, I. Ramesh Babu, Emily Ang, Lin Wenwei, Sylvie Alonso, Peter C. Dedon

**Affiliations:** 1 Department of Chemistry, 56-787, Massachusetts Institute of Technology, 77 Massachusetts Avenue, Cambridge, MA 02139, USA; Email: tszchan@MIT.EDU (C.T.Y.C.); 2 Department of Biological Engineering, 56-787, Massachusetts Institute of Technology, 77 Massachusetts Avenue, Cambridge, MA 02139, USA; Email: kslim@mit.edu (K.S.L.); rbabu@mit.edu (R.B.); 3 Singapore-MIT Alliance for Research and Technology, Center for Life Sciences #05-06, 28 Medical Drive, Singapore 117456, Singapore; Email: chia-hua.maggie.ho@smart.mit.edu (C.-H.H.); 4 Department of Microbiology, National University of Singapore, #03-05 28 Medical Drive, Singapore 117456, Singapore; Email: yh_chionh@nus.edu.sg (Y.H.C.); micaly@nus.edu.sg (E.A.); miclww@nus.edu.sg (L.W.); sylvie_alonso@nuhs.edu.sg (S.A.)

**Keywords:** tRNA, ribonucleoside modification, *Mycobacteriaum bovis*, BCG

## Abstract

There are more than 100 different ribonucleoside structures incorporated as post-transcriptional modifications, mainly in tRNA and rRNA of both prokaryotes and eukaryotes, and emerging evidence suggests that these modifications function as a system in the translational control of cellular responses. However, our understanding of this system is hampered by the paucity of information about the complete set of RNA modifications present in individual organisms. To this end, we have employed a chromatography-coupled mass spectrometric approach to define the spectrum of modified ribonucleosides in microbial species, starting with *Mycobacterium bovis* BCG*.* This approach revealed a variety of ribonucleoside candidates in tRNA from BCG, of which 12 were definitively identified based on comparisons to synthetic standards and 5 were tentatively identified by exact mass comparisons to RNA modification databases. Among the ribonucleosides observed in BCG tRNA was one not previously described in tRNA, which we have now characterized as *N^6^,N^6^*-dimethyladenosine.

## 1. Introduction

While the four canonical nucleobases in DNA are adequate to encode the genome, the functional diversity of RNA is greatly enhanced by the presence of over 100 different targeted structural modifications of the nucleobase and ribose sugar in RNA across all organisms [1,2]. Transfer RNA (tRNA) and ribosomal RNA (rRNA) are the most frequently modified forms of RNA, though other species such as messenger RNA (mRNA) and microRNAs are also known to possess specific ribonucleoside modifications, such as the N^7^-methylguanosine cap on mRNA.

Emerging evidence points to important roles for RNA modifications in translational control of cellular responses to stress and other stimuli [3,4,5,6]. For example, we recently observed that exposure of the yeast *Saccharomyces cerevisiae* to cytotoxic chemicals causes a reprogramming of the spectrum of two dozen RNA modifications in tRNA, with a unique ribonucleoside signature for each agent [7]. These observations point to the importance of fully understanding the structure and function of ribonucleoside analogs in both eukaryotic and prokaryotic organisms.

To this end, we have undertaken a systematic characterization of RNA modifications in a variety of pathological microorganisms, including mycobacteria. Infection with *Mycobacterium tuberculosis* (Mtb) represents one of the most widespread diseases in the world, with nearly one-third of the World's population showing signs of exposure, more than 20 million people actively infected, and almost 80% of the population of some developing countries testing positive in tuberculin tests [8,9]. *Mycobacterium bovis* Bacille Calmette-Guérin (BCG) is a mycobacterial species closely related to Mtb and is widely used as a vaccine [10]. To understand the biological roles of tRNA modifications in mycobacteria, we have begun to characterize the RNA modifications in BCG, with the discovery of *N^6^*,*N^6^*-dimethyladenosine in mycobacterial tRNA.

## 2. Results and Discussion

### 2.1. Isolation of tRNA

**Figure 1:**
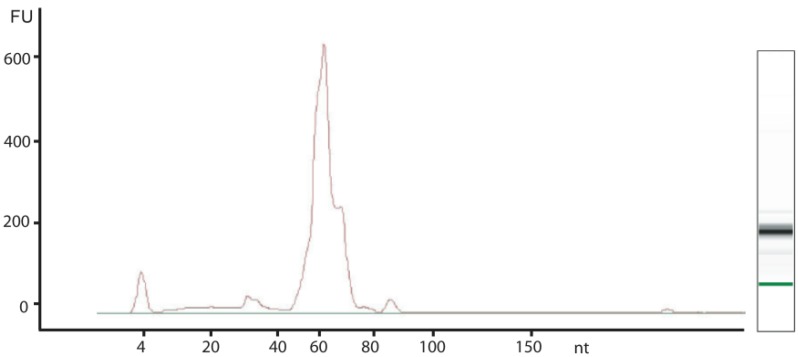
Characterization of BCG small RNA species. An aliquot of small RNA isolated from BCG was analyzed on an Agilent Bioanalyzer small RNA chip. The peak at 4 nt in the electropherogram represents a size standard; the image on the right is the reconstructed gel image of the resolved RNA species.

As the spectrum of modified nucleosides may vary significantly under different cell culture conditions and different stages of cell growth, we adopted a standard approach to BCG culture conditions by harvesting cells in log-phase growth at an OD_600_ of ~0.6, which occurred on day ~7 under our culture conditions. The procedure used to isolate small RNA species yielded ~4 μg of high quality tRNA from 10^9^ BCG cells. As shown in Figure 1, tRNA represented >95% of the RNA present in the sample. The average size of the major RNA peak was determined to be ~65 nt, which is consistent with the predicted size of tRNA in BCG [11]. One important issue relevant to these studies was the possibility of contamination of tRNA with 5S rRNA. In BCG, 5S rRNA is 115 nt in length [12] and there were no detectable RNA species of this size in the small RNA isolates prepared for the present studies (Figure 1).

### 2.2. Identification of Ribonucleosides in BCG tRNA

As a critical feature of our platform for studying the complete set of ribonucleosides in an organism [7], liquid chromatography-coupled mass spectrometry has previously been demonstrated to be a powerful tool for characterizing the structure of ribonucleosides and for quantifying them in biological systems [13,14]. Ribonucleosides from hydrolyzed tRNA were initially characterized using LC-MS/MS with neutral loss analysis. During CID, there is characteristic cleavage of the glycosidic bond between the nucleobase and either the ribose or 2’-*O*-methylribose moiety, which causes a loss of either 132 or 146 *amu*, respectively. We used this property to search for modified nucleosides, with parallel analysis of a sample prepared without added RNA to account for artifacts. It should be noted that, since the C–C glycosidic bond in pseudouridine (Y) does not readily fragment to produce neutral loss of a ribose residue, the presence of Y was verified by CID fragmentation of the ribose to yield the nucleobase with the ribose C1 methylene group attached (*m/z* 125; [15]).

**Figure 2:**
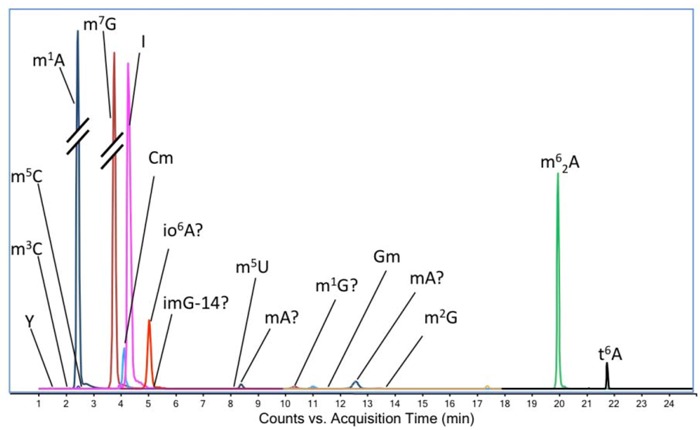
Extracted ion chromatogram of ribonucleoside candidates in hydrolyzed BCG tRNA identified by LC-MS/MS in neutral loss mode. The peaks of m^1^A (2.5 min) and m^7^G (3.9 min) are marked with ‘//’ to indicate that they are in different scales from other peaks. The identities of ribonucleosides marked with ‘?’ are tentative and have not been confirmed against standards. ‘mA’ denotes a monomethylated adenosine.

This analysis revealed several ribonucleoside candidates (Figure 2). Of these, 12 were definitively identified by comparison of retention time, exact mass and CID fragmentation to synthetic standards, while another five were tentatively identified on the basis of exact mass comparisons to RNA modification databases [1,2], with no specific structures that can be assigned to the isobaric methylated ribonucleosides (Table 1).

### 2.3. Structural Characterization of the Ribonucleoside with m/z 296.1350

**Figure 3:**
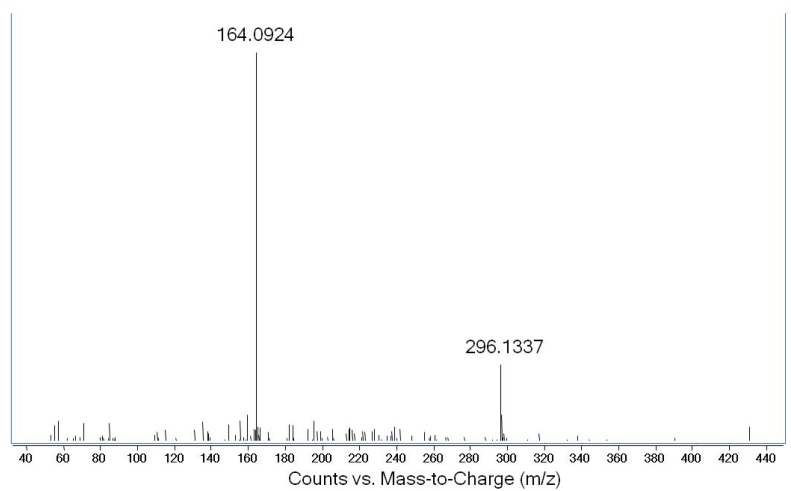
MS^2^ fragmentation of the ribonucleoside with *m/z* 296.1350.

**Figure 4:**
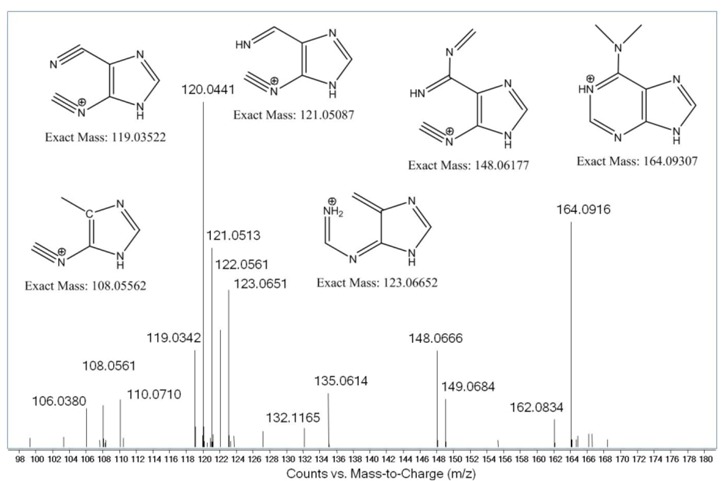
Pseudo-MS^3^ fragmentation of *m/z* 164.0924 derived from the *m/z* 296.1350 ribonucleoside. Tentative structures of fragment ions are based on a model proposed by Nelson and McCloskey [18].

**Figure 5:**
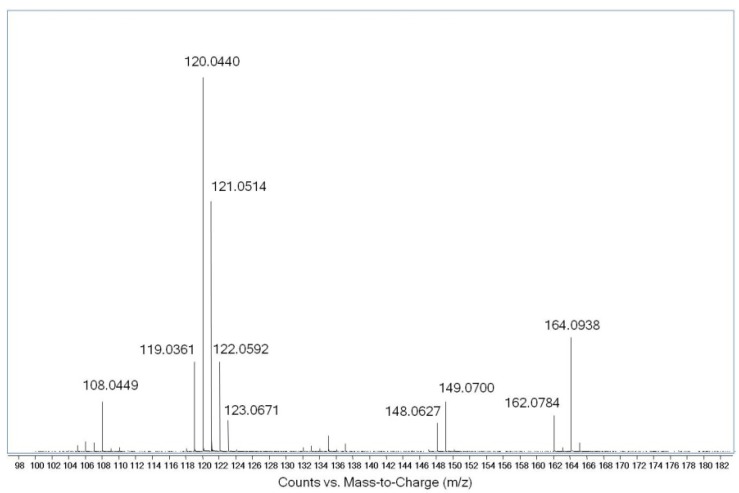
Pseudo-MS^3^ fragmentation of *m/z* 164.0924 derived from the *m/z* 296.1350 ion of synthetic m^6^_2_A.

One of the ribonucleosides identified in neutral loss analysis had a *m/z* value of 296.1350 ± 0.0011 (mean ± SD; Figures S1 and S2), which yields a chemical formula of C_12_H_18_N_5_O_4_^+^ (*m/z* 296.1359). This formula corresponded to an adenosine with 2 methyl groups or 1 ethyl group. Subsequent MS^2^ analysis confirmed the neutral loss of ribose to yield a fragment with *m/z* 164.0924 (Figure 3), which corresponds to an adenine nucleobase with an additional C_2_H_4_ (*m/z* 164.0936). Pseudo-MS^3^ analysis consisted of in-source fragmentation-induced loss of ribose to yield an ion with *m/z* 164.09, which was selected for CID in the second quadrupole. The resulting mass spectrum shown in Figure 4 revealed a variety of fragment ions, many of which had *m/z* values associated with fragmentation of methylated adenosine [16]. Based upon this dissociation model and the fragmentation pattern, we concluded that the structure of the ion with *m/z* 296.1350 was *N^6^,N^6^*-dimethyladenosine (m^6^_2_A). This was confirmed by comparison to synthetic m^6^_2_A, which produced identical values for retention time, exact mass, and MS^2^ and pseudo-MS^3^ fragmentation (Figure 5).

**Table 1:** Ribonucleosides identified by mass spectrometric analysis of BCG tRNA hydrolysates ^1^.

Retention time, min ^1^	Precursor ion, *m/z*	Product ion, *m/z*	Signal Intensity	Identity ^2^
1.43	245.1	125.1	110	**Y**
2.15	258.1	126.1	70	**m^5^C**
2.45	258.1	126.1	279	**m^3^C**
2.47	282.1	150.1	500000	**m^1^A**
3.85	298.1	166.1	500000	**m^7^G**
4.22	258.1	112.1	2500	**Cm**
4.4	269.1	137.1	80000	**I**
5.16	352	220	16000	io^6^A?
5.27	322	190	24000	imG-14?
8.22	259.1	127.1	30	**m^5^U**
8.6	282.1	150.1	7000	mA, Am?
10.69	298.1	166.1	3500	m^1^G?
11.4	298.1	152.1	450	**Gm**
12.9	282.1	150.1	17000	mA, Am?
13.91	298.1	166.1	600	**m^2^G**
20.1	296.1	164.1	25000	**m^6^_2_A**
21.88	413.1	281.1	2500	**t^6^A**

^1^ Neutral loss analysis (except for Y) was performed with a triple quadrupole mass spectrometer, as described in the Experimental section; ^2^ RNA modifications noted in bold font were corroborated with synthetic standards. “?” denotes tentative identification; “mA, Am” denotes a monomethylated adenosine.

### 2.4. Quantification of m^6^_2_A in tRNA from Different Organisms

Since m^6^_2_A had been described in rRNA previously but not in tRNA, we quantified this modification by LC-MS/MS in samples of tRNA from *M. bovis* BCG, rat liver tissue, TK6 cell line, and yeast by using external calibration against dA (Figures S3 and S4). In *M. bovis* BCG, the level of m^6^_2_A was 0.88 pmol per μg of tRNA, while the level of m^6^_2_A in rat liver, and TK6 and yeast cells was below the detection limit of the assay. On average, the size of mycobacterial tRNA is ~65 nt, which suggests that m^6^_2_A occurs once in every ~51 tRNA molecules. The data in Table 2 also suggest that the presence of m^6^_2_A in the small RNA isolates is not due to contamination with rRNA fragments, since we would have expected to detect m^6^_2_A in the small RNA isolates from the other species if rRNA fragment contamination had occurred. To further confirm this, tRNA was purified from samples of BCG small RNA by size-exclusion HPLC (Figure S5) and m^6^_2_A was again detected in the fraction containing only tRNA (data not shown).

**Table 2:** Level of m^6^_2_A in tRNA from BCG, human, rat and yeast.

	BCG	Human TK6	Rat liver	*S. cerevisiae*
MS signal^1^	5.8 ± 0.9	<0.006^2^	<0.006^2^	<0.006^2^
pmol/μg tRNA	0.88 ± 0.14	< 0.0009^2^	< 0.0009^2^	< 0.0009^2^

^1^ MS signal normalized to total tRNA (0.4 μg); ^2^ Less than the limit of quantification.

### 2.5. Discussion

Using an LC-MS platform developed for analysis of ribonucleoside modifications in yeast [7], we have begun to systematically characterize the spectrum of modified ribonucleosides in a variety of microorganisms, starting with *Mycobacterium bovis* BCG. The platform that we have developed addresses all levels of highly quantitative analysis, including isolation of high quality RNA (Figure 1), rigorous quantification of the RNA, and RNA processing under conditions that obviate modification artifacts caused by adventitious oxidation (addition of deferoxamine and butylated hydroxytoluene) and deamination (addition of coformycin and tetrahydrouridine). Subsequent resolution of the complete set of ribonucleosides by reversed-phase HPLC provides good separation (Figure 2) for mass spectrometric characterization for both high mass accuracy and fragmentation. Using this approach, we were able definitively identify 12 modified ribonucleosides in BCG tRNA: Y, m^5^C, m^3^C, m^1^A, m^7^G, Cm, I, m^5^U, Gm, m^2^G, t^6^A, and m^6^_2_A. All of these species have been described previously in either tRNA or rRNA from other organisms [17,18], which suggests conservation of function in mycobacteria. Of these ribonucleosides, only 1-methyladenosine (m^1^A) has been previously identified in a mycobacterial species (*Mtb)*, where it occurs at position 58 of tRNA [18,19]. While m^1^A and m^7^G are highly abundant in both BCG and yeast tRNA, the relative proportions of other ribonucleosides common to both organisms differ significantly, as judged from mass spectrometric signal strength under identical analytical conditions (Figure 2 in the present studies and Figure 1 in [7]).

In addition to the 12 defined ribonucleosides, we tentatively identified five other ribonucleosides on the basis of CID molecular transitions and exact mass comparisons with RNA modification databases: *N^6^*-(cis-hydroxyisopentenyl)adenosine (io^6^A; *m/z* 352.1616; *m/z* 352→220), 4-demethylwyosine (imG-14; *m/z* 322.1146; *m/z* 322→190), two adenosine species in which the nucleobase is methylated (*m/z* 282.1197; *m/z* 282→150) and a species in which the guanosine nucleobase is methylated (*m/z* 298.1146; *m/z* 298→166). The latter is likely to be m^1^G since no other mono-methylated guanosines than Gm, m^2^G and m^7^G have been described [19,20]. Possible candidates for mono-methylated adenosine include Am, m^2^A m^8^A, m^6^A; m^7^A has not been described previously [17,18]. These modifications have been observed in tRNA in other organisms [17,18].

Our initial analysis of ribonucleosides in BCG tRNA revealed an abundant (Figure 2) ribonucleoside not previously identified in tRNA: an adenosine species with either an ethyl group or two methyl groups attached to the nucleobase. Among the possible candidates for this species were 1,2-*O*-dimethyladenosine (m^1^Am), N^6^,2-*O*-dimethyladenosine (m^6^Am) and N^6^,N^6^-dimethyladenosine (m^6^_2_A) [17,18]. As shown in Figure 3 and Figure 4, MS^2^ and pseudo-MS^3^ fragmentation suggested that the structure was m^6^_2_A, a conclusion corroborated by comparison to a synthetic m^6^_2_A standard (Figure 5). The nucleoside m^6^_2_A was discovered by Littlefield and Dunn in *Bacterium coli*, *Aerobacter aerogenes*, yeast, and rat liver tissues [20]. It was identified in the 16S and 23S rRNA of archaea and bacteria and the 18S rRNA of eukarya [21,22,23,24,25]. However, m^6^_2_A has not been identified in tRNA from any organism or cell types other than BCG, including yeast, rat liver and human cells (Table 2). That the m^6^_2_A did not arise from contamination of small RNA isolates with 5S rRNA or fragments of larger rRNA species is supported by the results in Table 2, the Bioanalyzer results in Figure 1 and Figure S4, and the analysis of HPLC-purified tRNA from BCG.

While m^6^_2_A has been observed previously only in rRNA, several features of its structure and biosynthesis may provide insights into its presence in tRNA. An examination of ribonucleoside databases reveals that adenosine species with *N^6^* modifications (e.g., t^6^A, i^6^A) tend to be located at position 37 of tRNA, which suggests a possible location for the similarly hydrophobic m^6^_2_A in tRNA in BCG and other mycobacterial species. Orthologs of the methyltransferase KsgA catalyze the formation of m^6^_2_A in the 3’-ends of the small subunit rRNAs in most organisms [26], while members of the Erm family of methyltransferases catalyze m^6^_2_A formation in 23S rRNA in many bacteria [27,28]. Homologs of both Erm and KsgA are present in BCG and *Mtb* [12,29]. Given the precedent for m^5^U formation in both tRNA and 16S rRNA by the tRNA (m^5^U54) methyltransferase [30,31], it is possible that the KsgA or Erm homologs also catalyze m^6^_2_A formation in BCG tRNA.

## 3. Experimental

### 3.1. Materials and Equipment

All chemicals and reagents were of the highest purity available and were used without further purification. OADC solution, 7H9 culture media powder, and 7H11 agar powder were purchased from Biomed Diagnostics (White City, OR, USA). Trizol reagent and PureLink miRNA Isolation Kit was purchased from Invitrogen (Carlsbad, CA, USA). 2’-*O*-Methyluridine (Um), pseudouridine (Y), *N^1^*-methyladenosine (m^1^A), *N^2^*,*N^2^*-dimethylguanosine (m^2^_2_G), *N^6^,N^6^*-dimethyladensoine (m^6^_2_A), and 2’-*O*-methylguanosine (Gm) were purchased from Berry and Associates (Dexter, MI, USA). *N^6^*-threonyl-carbamoyladenosine (t^6^A) was purchased from Biolog (Bremen, Germany). *N^6^*-isopentenyladenosine (i^6^A) was purchased from International Laboratory LLC (San Bruno, CA, USA). 2’-*O*-Methyladenosine (Am), *N^4^*-acetylcytidine (ac^4^C), 5-methyluridine (m^5^U), inosine (I), 2-methylguanosine (m^2^G), *N^7^*-methylguanosine (m^7^G), 2’-*O*-methylcytidine (Cm), 3-methylcytidine (m^3^C), 5-methylcytidine (m^5^C), alkaline phosphatase, RNase A, ammonium acetate, geneticine, bovine serum albumin, deferoxamine mesylate, butylated hydroxytoluene, glucose, sodium chloride, nuclease P1, formic acid, and 20% Tween80 solution were purchased from Sigma Chemical Co. (St. Louis, MO, USA). Glycerol was purchased from SinoChem Corp. (Beijing, China). Phosphodiesterase I was purchased from USB (Cleveland, OH, USA). RNase A, RNase V1 and RNase T1 were purchased from Ambion Inc. (Austin, TX, USA). HPLC-grade water, acetonitrile, and chloroform were purchased from Mallinckrodt Baker (Phillipsburg, NJ, USA). *M. bovis BCG* and *S. cerevisiae* were purchased from American Type Culture Collections (Manassas, VA, USA). Rat liver tissue (discarded) was obtained from Laura J. Trudel (Department of Biological Engineering, Massachusetts Institute of Technology). Filters with a 10 KDa MW cut-off were purchased from Pall Life Sciences (Port Washington, NY, USA). Experiments were performed with Thermo FP120 Bead beater (Two Rivers, WI, USA), Qiagen TissueRuptor (Valencia, CA, USA), Agilent Bioanalyzer series 2100, Agilent LC/QQQ 6460, Agilent LC/TOF G6210A, and Agilent LC/QTOF 6520 (Santa Clara, CA, USA).

### 3.2 Culturing BCG

*Mycobacterium bovis* BCG cells were grown in 7H9 culture media (see Supporting Information) at 37 °C in an incubator with 5% CO_2_. After the culture reached an optical density of OD_600_ ~0.6, at which point the concentration of cells was ~3 × 10^7^/mL, the cells were harvested by centrifugation at 12,000 × g for 10 min at 4 °C. Cell pellets were snap-frozen in liquid nitrogen and stored at −80 °C. For glycerol stocks, the post-centrifugation cell pellet was resuspended in 1 mL of 7H9 media with 25% glycerol. The solution was then further diluted to a final concentration with OD_600_~1 and the stocks stored at −80 °C. To determine the quantity of living cells in a glycerol stock or a culture, colony counting was performed for each cell sample with 100 μL of serial dilutions plated on 7H11 agar and incubated at 37 °C with 5% CO_2_ (see Supporting Information).

### 3.3. Isolation of tRNA

tRNA was isolated from several organisms, including BCG (~10^9^ cells), *S. cerevisiae* (5 × 10^7^ cells), human B lymphoblastoid TK6 cells (3 × 10^7^ cells), and rat liver (~150 mg). Cells or tissues were suspended in 1.5 mL of Trizol reagent with 5 mg/mL coformycin, 50 μg/mL tetrahydrouridine, 0.1 mM deferoxamine mesylate, and 0.5 mM butylated hydroxytoluene to prevent nucleoside modification artifacts [32,33]. BCG and *S. cerevisiae* cells were lysed by 3 cycles of bead beating in a Thermo FP120 Bead Beater set at 6.5 m/s for each 20 s cycle, with 1 min of cooling on ice between cycles. The TK6 cells and rat liver tissue were lysed with a Qiagen TissueRuptor. Following cell or tissue disruption, all lysates were warmed to ambient temperature for 5 min and extracted with 0.3 mL volume of chloroform, with subsequent incubation at ambient temperature for 3 min. The solutions were centrifuged at 12,000 × g for 15 min at 4 °C and the aqueous phase was collected. Absolute ethanol was added to the aqueous phase (35% v/v) and small RNA species were then isolated using the PureLink miRNA Isolation Kit according to manufacturer’s instructions. The quality and concentration of the resulting small RNA mixture was assessed with a Bioanalyzer (Agilent Small RNA Kit), with tRNA comprising >95% of the small RNA species present in the mixture, with no detectable 5S rRNA (Figure 1). In one study, tRNA was purified from BCG small RNA isolates by size-exclusion HPLC using an Agilent SEC3 300 Å, 7.8 × 300 mm column eluted with 10 mM ammonium acetate. The tRNA fraction was collected and desalted using an Ambion Millipore 5K MWCO column.

### 3.4. Enzymatic Hydrolysis of BCG tRNA

Samples of purified RNA (6 μg) were lyophilized and redissolved in 100 μL of 10 ng/mL RNase A, 0.01 U/mL RNase T1, 0.001 U/mL RNase V1, 0.15 U/mL nuclease P1, 2.5 mM deferoxamine mesylate, 10 ng/mL coformycin, 50 μg/mL tetrahydrouridine, 0.5 mM butylated hydroxytoluene, and 1 × RNA Structure Buffer from Ambion. The solution was incubated at 37 °C for 3 h, after which alkaline phosphatase was added to a final concentration of 0.1 U/mL. The sample was incubated at 37 °C overnight, followed by removal of proteins by YM10 filtration. The resulting filtrate was used directly for mass spectrometric analysis.

### 3.5. Identification of Ribonucleosides in Small RNA from BCG

Hydrolyzed RNA was resolved on a Thermo Hypersil Gold aQ column (100 × 2.1 mm, 1.9 μm particle size) with an acetonitrile gradient (HPLC system A) in 0.1% (v/v) formic acid in water as mobile phase at a flow rate of 0.3 mL/min. The gradient of acetonitrile in 0.1% formic acid was as follows: 0–15.3 min, 0%; 15.3–18.7 min, 1%; 18.7–20 min, 6%; 20–24 min, 6%; 24–27.3 min, 100%; 27.3–41 min, 0%. The HPLC column was directly connected to a triple quadrupole mass spectrometer (Agilent LC/QQQ 6460) in positive ion, neutral loss mode for loss of *m/z* 132 and 146 in the range of *m/z* 200–700. The voltages and source gas parameters were as follows: Gas temperature, 300 °C; gas flow, 6 L/min; nebulizer, 15 psi; and capillary voltage, 4000 V. The ions detected in neutral loss mode were selected for identification with the LC-MS/MS system in multiple reaction monitoring (MRM) mode using the same HPLC and mass spectrometer parameters. Since the C–C glycosidic bond in pseudouridine (Y) does not readily fragment to produce neutral loss of a ribose residue, the presence of Y was verified by CID fragmentation of the ribose to yield the nucleobase with the ribose C1 methylene group attached (*m/z* 125; [15]).

### 3.6. High Mass-Accuracy Mass Spectrometric Analysis of Candidate Ribonucleosides

The exact molecular weights of candidate ribonucleosides were determined by HPLC-coupled high mass-accuracy quadrupole time-of-flight mass spectrometry (Agilent LC/QTOF 6520). Hydrolyzed RNA samples were resolved on a Thermo Hypersil Gold aQ column (150 × 2.1 mm, 3 μm particle size) eluted with a mobile phase as noted earlier with the following solvent schedule for acetonitrile in 0.1% formic acid: 0–23 min, 0%; 23–28 min, 2%; 28–36 min, 7%; 36–47 min, 100%; 47–67 min, 0% (HPLC system, B). The HPLC system was coupled to an Agilent QTOF 6520 Mass Spectrometer operated in positive ion mode scanning *m/z* 100–1700 with the following parameters: gas temperature, 325 °C; drying gas, 5 L/min; nebulizer, 30 psi; and capillary voltage, 3500 V.

### 3.7. Structural Characterization of N^6^,N^6^-Dimethyladenosine in BCG Small RNA

The ribonucleoside-like species eluting at 20.1 min and possessing an [M+H]^+^ ion with *m/z* of 296.1337 was subjected to structural characterization by collision-induced dissociation (CID) using both MS^2^ and pseudo-MS^3^ (*i.e.*, in-source fragmentation) performed on the LC-QTOF system using a Thermo Hypersil Gold aQ column (100 × 1 mm, 3 μm particle size) at a flow rate of 90 μL/min using the same mobile phase described earlier, with a solvent schedule for the 0.1% formic acid in acetonitrile as follows: 0–18 min, 0%; 18–30 min, 7%; 30–44 min, 100%; and 44–60 min, 0% (HPLC system C). The mass spectrometer was operated in positive ion mode with the following voltages and source gas parameters: gas temperature, 325 °C; drying gas, 8 L/min; nebulizer, 30 psi; capillary voltage, 3500 V. The *m/z* detection range for parent ions was 100 to 800 and that for product ions was 50 to 800. For MS^2^ analysis, the fragmentor voltage was 85 V, the collision energy was 10 V and the target ion for the unknown was *m/z* 296.1, while the fragmentor voltage was increased to 250 V for MS^3^ analysis, which caused an in-source fragmentation of *m/z* 296.1 to give *m/z* 164.1 for further CID analysis. The *m/z* 164.1 ion was fragmented with collision energies of 0 V, 20 V, 30 V, and 60V.

### 3.8. Quantification of N^6^,N^6^-Dimethyladenosine in tRNA from BCG and Other Organisms

Absolute quantification of *N^6^,N^6^*-dimethyladenosine (m^6^_2_A) was achieved by LC-MS/MS as described earlier, with [^15^N]_5_-2-deoxyadenosine ([^15^N]-dA) as the internal standard. For unknown tRNA samples, 4 pmol of [^15^N]-dA was added to 4 μg of tRNA and the samples were subjected to enzymatic hydrolysis as described earlier. Following volume adjustment to achieve final concentrations of ~40 nM [^15^N]-dA and ~40 ng/μL ribonucleosides, 10 μL of sample was analyzed by LC-MS/MS. Ribonucleosides were resolved on a Thermo Hypersil Gold aQ column (100 × 2.1 mm, 1.9 μm particle size) with 0.1% (v/v) formic acid in water and in acetonitrile as mobile phase and a flow rate of 0.3 mL/min. The solvent schedule for acetonitrile in 0.1% formic acid was as follows: 0–10 min, 5%; 10–12 min, 30%; 12 min, 95%. Electrospray ionization MS/MS analysis of the HPLC eluant was performed in positive ion mode with the following parameters for voltages and source gas: Gas temperature, 350 °C; gas flow, 10 L/min; nebulizer, 20 psi; and capillary voltage, 3500 V. The mass spectrometer was operated in MRM mode to quantify two ribonucleosides with the following parameters (retention time, *m/z* of the transmitted parent ion, *m/z* of the monitored product ion, fragmentor voltage, collision energy): [^15^N]-dA—3.0 min, *m/z* 257→141, 90 V, 10 V; and m^6^_2_A—11.1 min, *m/z* 296→164, 90 V, 15 V. The dwell time for each ribonucleoside was 200 ms and these two ions were monitored throughout the HPLC run. Linear calibration curves were obtained using a fixed concentration (40 nM) of [^15^N]-dA and varying concentrations of m^6^_2_A (5, 10, 50, 100, 500 nM).

## 4. Conclusions

We have employed a chromatography-coupled mass spectrometric approach to systematically define the spectrum of modified ribonucleosides in *Mycobacterium bovis* BCG*.* This approach revealed a variety of ribonucleoside candidates in tRNA from BCG, of which 12 were definitively identified based on comparisons to synthetic standards and 5 were tentatively identified by exact mass comparisons to RNA modification databases. Among the ribonucleosides observed in BCG tRNA was one not previously described in tRNA, which we have now characterized as *N^6^,N^6^*-dimethyladenosine.

## Supplementary Materials

Supplementary Materials can be accessed at http://www.mdpi.com/1420-3049/16/6/5168/s1.
