# Supplementary material for: Identification of N6,N6-Dimethyladenosine in Transfer RNA from Mycobacterium bovis Bacille Calmette-Guérin
Source: Molecules. 2011 Jun 21;16(6):5168–81. doi: 10.3390/molecules16065168 (PMC6264175; doi:10.3390/molecules16065168)

Molecules **2011**, *16*

molecules

ISSN 1420-3049

www.mdpi.com/journal/molecules

Supplementary Materials

Methods

Preparation of BCG culture media. The 7H9 media were prepared by mixing 4.9 g of 7H9 powder, 10 mL of 50% glycerol, 2.5 mL of 20% TWEEN 80, 900 mL of double-deionized water, and 100 mL of ADS solution. The ADS solution was prepared by mixing 50 g of BSA, 20 g of glucose, and 8.1 g of NaCl in 950 ml of double-deionized water. The 7H11 agar plates were prepared by mixing 4.2 g of 7H11 agar powder, 2 mL of 50% glycerol, 180 ml of double-deionized water, and 20 mL of OADC solution. The solution was then heated in a microwave oven until a clear solution was achieved and the solution was transferred to petri dishes. The agar plates were cooled and solidified at ambient temperature.

Figures

Figure S1. Extracted ion chromatogram of ions with *m/z* 296.13 from the LC/TOF scan of the hydrolyzed tRNA.

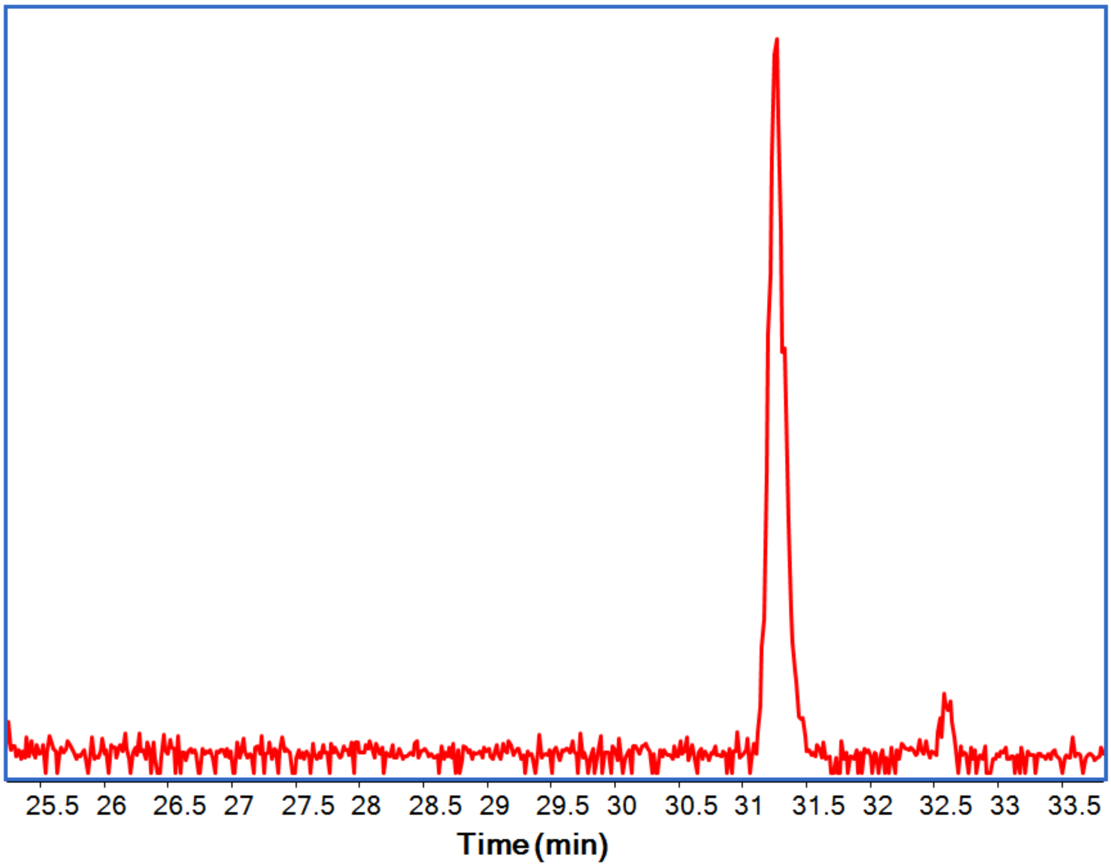

Figure S2. A background-subtracted mass spectrum at time = 31.3 min from Figure S1.

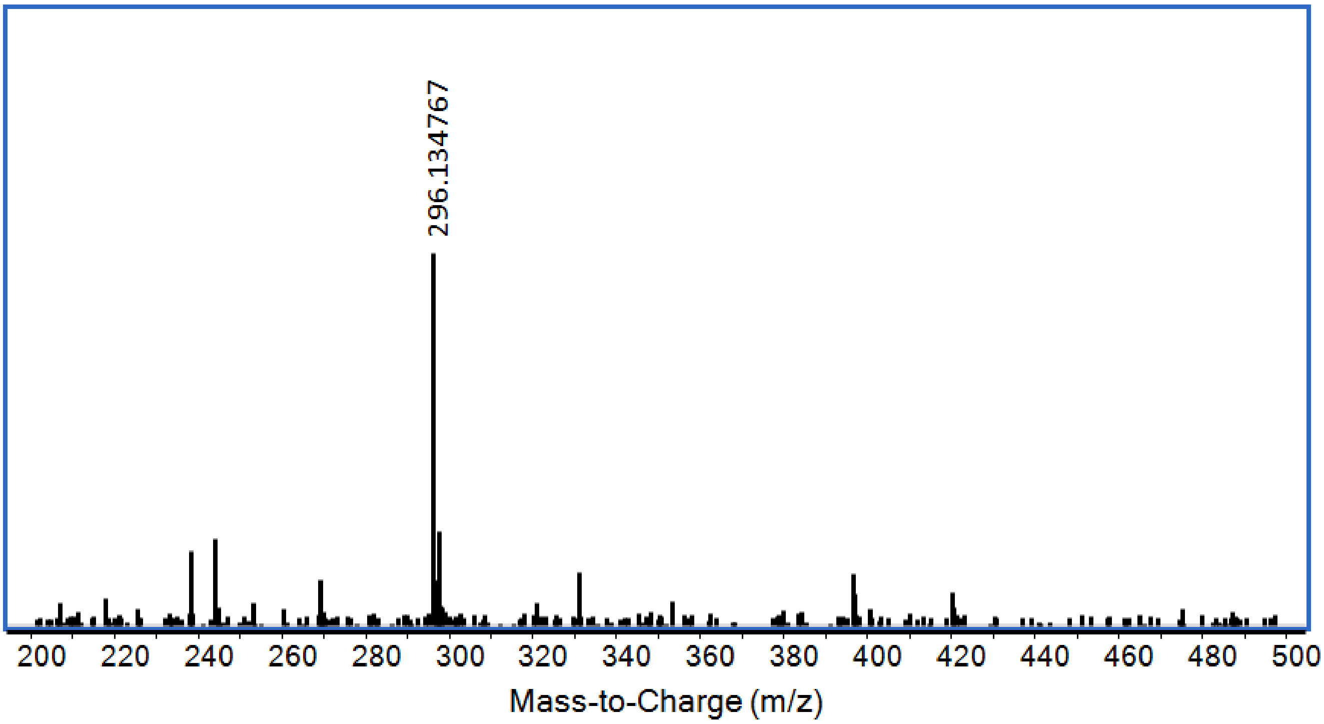

Figure S3. External calibration curve for quantifying $m^6{}_2A$ as described in the Experimental section.

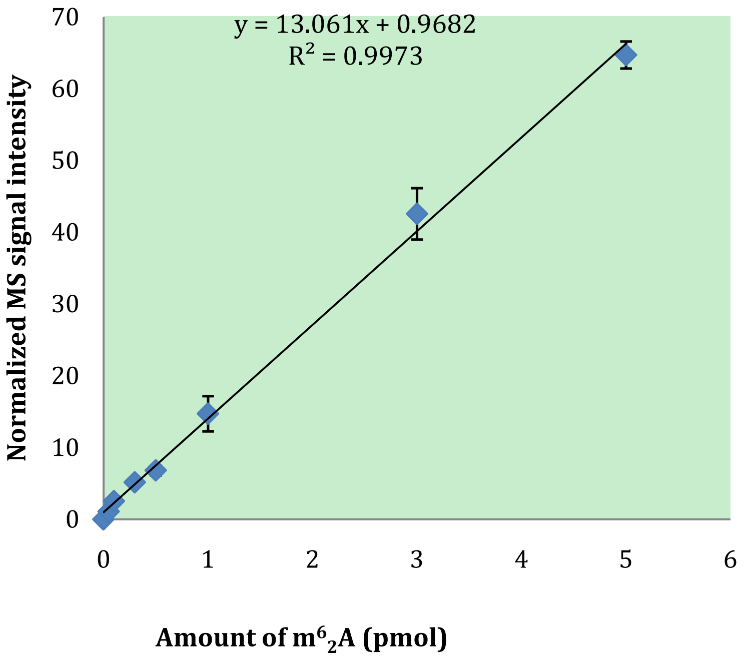

Figure S4. Analysis of small RNA isolated from the yeast *S. cerevisiae*, rat liver, and human B lymphoblastoid TK6 cells (respectively, from top to bottom). Samples were analyzed with an Agilent Bioanalyzer as described in the legend on Figure 1.

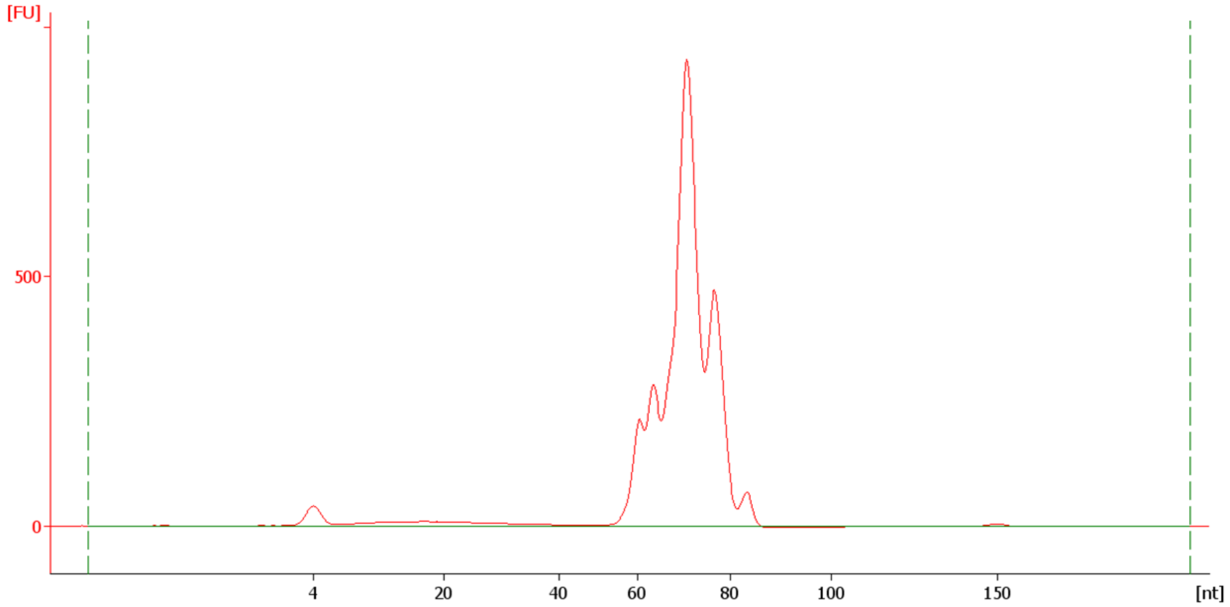

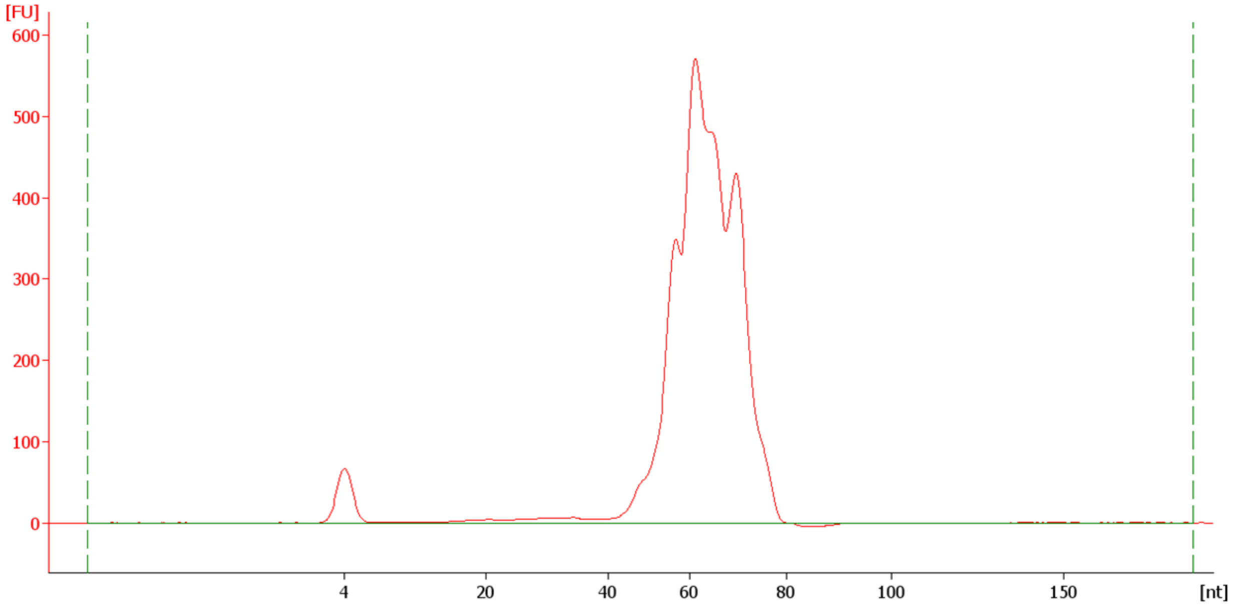

Figure S4. *Cont.*

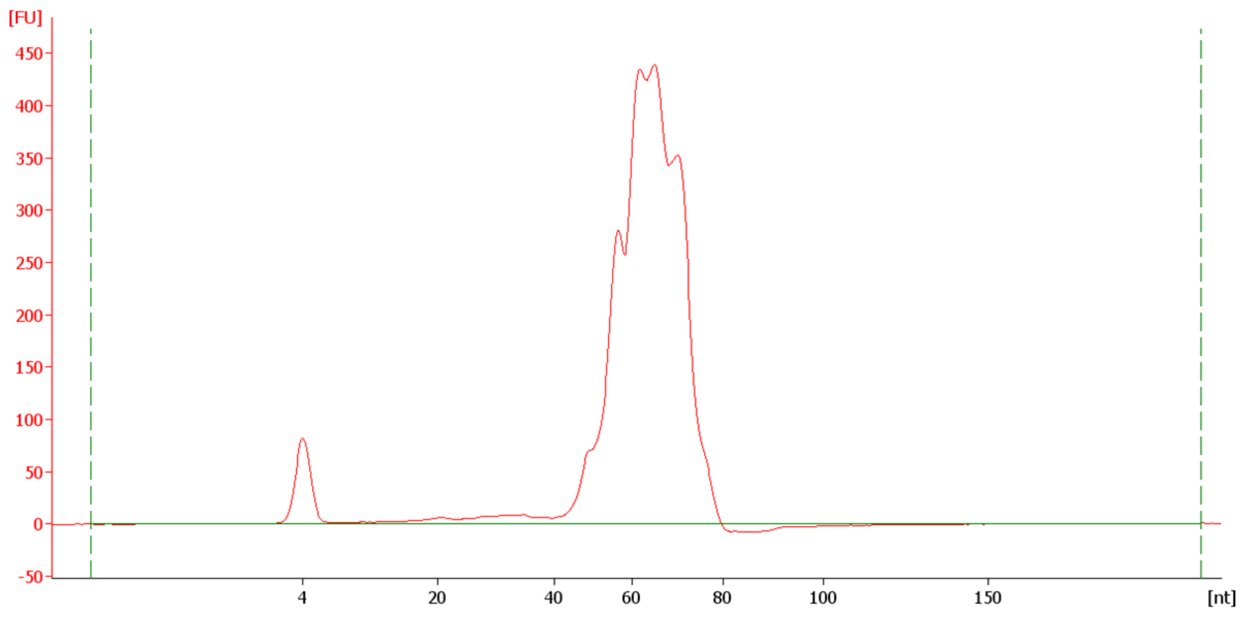

Figure S5. Purification of BCG tRNA from small RNA isolates by size-exclusion HPLC. Fraction A was collected and the tRNA analyzed for m^6_2A content as described in the text.

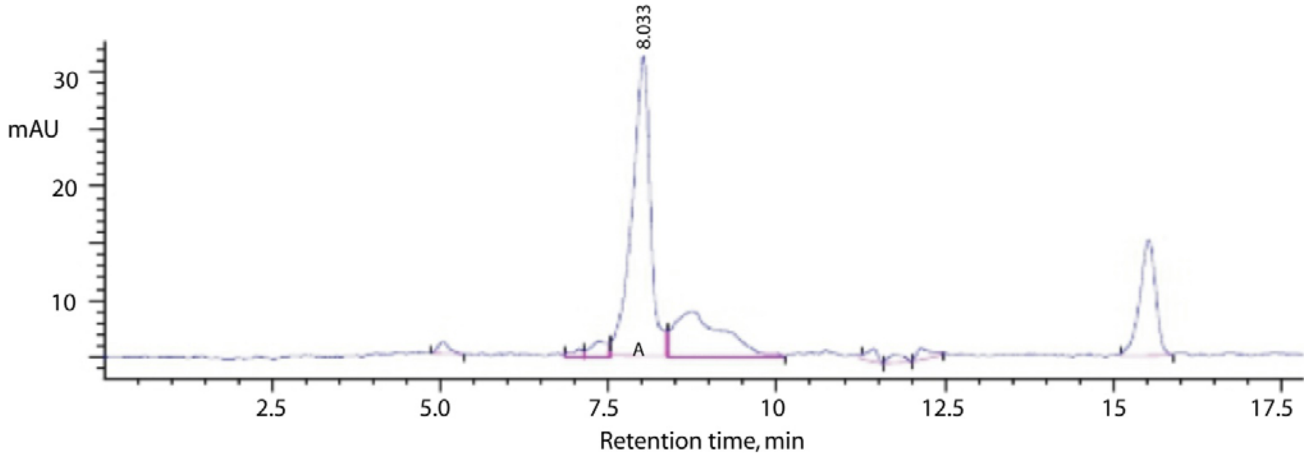

Supplement: Supplementary file 1 [file molecules-16-05168-s001.pdf]
